# Maize Varieties Released in Different Eras Have Similar Root Length Density Distributions in the Soil, Which Are Negatively Correlated with Local Concentrations of Soil Mineral Nitrogen

**DOI:** 10.1371/journal.pone.0121892

**Published:** 2015-03-23

**Authors:** Peng Ning, Sa Li, Philip J. White, Chunjian Li

**Affiliations:** 1 Department of Plant Nutrition, China Agricultural University, Beijing, China; 2 Ecological Sciences, The James Hutton Institute, Invergowrie, Dundee, United Kingdom; University of Michigan, UNITED STATES

## Abstract

Larger, and deeper, root systems of new maize varieties, compared to older varieties, are thought to have enabled improved acquisition of soil resources and, consequently, greater grain yields. To compare the spatial distributions of the root systems of new and old maize varieties and their relationships with spatial variations in soil concentrations of available nitrogen (N), phosphorus (P) and potassium (K), two years of field experiments were performed using six Chinese maize varieties released in different eras. Vertical distributions of roots, and available N, P and K in the 0–60 cm soil profile were determined in excavated soil monoliths at silking and maturity. The results demonstrated that new maize varieties had larger root dry weight, higher grain yield and greater nutrient accumulation than older varieties. All varieties had similar total root length and vertical root distribution at silking, but newer varieties maintained greater total root length and had more roots in the 30–60 cm soil layers at maturity. The spatial variation of soil mineral N (N_min_) in each soil horizon was larger than that of Olsen-P and ammonium-acetate-extractable K, and was inversely correlated with root length density (RLD), especially in the 0–20 cm soil layer. It was concluded that greater acquisition of mineral nutrients and higher yields of newer varieties were associated with greater total root length at maturity. The negative relationship between RLD and soil N_min_ at harvest for all varieties suggests the importance of the spatial distribution of the root system for N uptake by maize.

## Introduction

The development of new varieties, together with improved management practices, has played an important role in increasing grain yields of maize [1]. New maize varieties invest greater biomass in their root systems than older varieties, which can facilitate the acquisition of soil resources and, thereby, support greater shoot biomass and increase crop yield [2, 3]. However, the effective acquisition of water and nutrients by a root system depends not only on its size but also on its length/biomass quotient and the three-dimensional distribution of roots in the soil [4–8]. It has yet to be determined whether the larger biomass of new maize varieties equates with greater total length of the root system and whether there are any differences in root length density (RLD) in the soil volume between older maize varieties and new ones with greater yields.

Widely extended lateral roots and branching angles are important for the acquisition of mineral nutrients. The optimal lateral root branching density in the maize root system depends on the relative availability of nitrate and P [4, 8–11]. In a field experiment with ten maize cultivars, root length densities and nitrate depletion in the subsoil were positively correlated at subsoil layer [12], possibly because nitrate can be leached into the deep soil layer in the wet season, and deeper roots are beneficial for the acquisition of nitrate at depth [9, 13, 14]. Maize root systems display great plasticity in their response to soil nitrate distribution, and lateral roots can proliferate in deep nitrate-rich soil horizons in the wet season, even at the reproductive stage [14]. A steep and deep root system allows maize to acquire water at depth, as well as leached nitrate [9]. Hammer et al. [15] modeled the relationship between root architecture and grain yield of maize released in U.S. Corn Belt and concluded that changes in root system architecture and water capture through breeding have had a direct effect on biomass accumulation and historical yield trends. Apparently, the deeper roots of more recent varieties enable plants to access water in deep soil horizons and, thereby, reduce risks of drought stress [6, 15]. However, whether newer maize varieties have steeper and deeper root systems than older ones still needs to be determined.

Increased nutrient uptake by plants can be achieved through the proliferation of roots in regions of the soil where nutrients are present, and thorough the acceleration of nutrient movement in the soil solution to the root surface [16, 17]. The majority of the nitrogen (N), phosphorus (P) and potassium (K) acquired by plants is delivered to the root surface by mass flow of the soil solution and diffusion in the rhizosphere [17, 18]. The mass flow of water and dissolved solutes to the root surface is determined by differences in water potential, soil hydraulic conductivity and shoot transpiration [19, 20]. Nitrate is present at high concentrations in the soil solution and has a large diffusion coefficient. By contrast, phosphate has a low solubility and low diffusion coefficient in soil solutions. Although K can be present at high concentrations in soil solutions, it is often strongly adsorbed to the soil matrix [16, 21, 22]. Nitrate is often assumed to move to the root surface largely by mass flow of the soil solution, while K and P are delivered mainly by diffusion [17, 18]. Because of the large differences in effective diffusion coefficients of nitrate, K and P in the soil, the calculated slope of the depletion curve of P in the rhizosphere is very steep, while that of nitrate is very gentle, and that of K is in the middle [18]. It is, therefore, predicted that on the single root basis, the gradient in nitrate concentration at the root surface relative to that in the bulk soil solution will be less than that of P and K [18]. Under the field condition, however, the spatial variations of soil available nutrients in different soil horizons and especially their relationship with root length density of maize are still unknown.

The objectives of the present work are (1) to compare the changes in root spatial distribution in the soil profile under field-grown maize released in different eras; and (2) to clarify the spatial variations of the concentrations of soil available N, P and K in different soil horizons and their relationship with root length distribution of maize grown under field conditions. To address these questions, field experiments were performed in two years using six Chinese maize varieties released in different eras. Vertical root distribution, expressed as RLD, in the 0–60 cm soil profile of excavated soil monoliths, was measured at silking and at maturity and compared with the spatial distribution of soil mineral N (N_min_), Olsen-P and ammonium acetate (NH_4_OAc) extractable K.

## Materials and Methods

### Experimental design

The field experiments were performed in 2008 and 2009 at two adjacent sites at the Shangzhuang Experimental Station (40^o^8’20”N, 116^o^10’47”E), China Agricultural University, Beijing, China. The soil is a typical Ustochrept with a silty loam texture, mesic soil temperature regime, and mixed mineralogy. The soil was sampled using an auger and analyzed before sowing in both years. The chemical properties of the 0–30 cm soil layer of the field sites in 2008 and 2009 were: CaCl_2_ extractable mineral N (N_min_) 8.0 and 7.7 mg kg^−1^, pH (H_2_O) 8.0 and 7.9, Olsen-P 7.6 and 7.1 mg kg^−1^, NH_4_OAc extractable K 86.3 and 97.6 mg kg^−1^, and organic matter 10.5 and 7.3 g kg^−1^, respectively. A few days before plowing and sowing, the field was irrigated sufficiently with a sprinkler in both years.

Six Chinese maize varieties released in the last 60 years were used in 2009: Baimaya (BMY) and Jinhuanghou (JHH), two open-pollinated varieties released in the 1950s; Zhongdan 2 (ZD2) and Tangkang 5 (TK5), two hybrids released in the 1970s; Nongda 108 (ND108) and Zhengdan 958 (ZD958), two currently popular hybrids. They show early senescing, intermediate senescing and stay green traits, respectively. New varieties (ND108 and ZD958) had larger total leaf area and longer green leaf duration, took up more N, P and K, had higher grain yield and nutrient use efficiency than old varieties (BMY and JHH) [23]. In 2008, only Baimaya, Zhongdan 2, Tangkang 5 and Zhengdan 958 were studied. Seeds were sown on 8 May 2008 and 20 May 2009. The plots were over-seeded (two seeds in each point) with hand planters and then thinned at the seedling stage to a stand of 60,000 plants ha^−1^. The experiments employed a randomized block design with three replicates, and each plot was 6 m long and 4.8 m wide. The distances between rows and plants within rows were 60 cm and 28 cm, respectively. Border plots were included at the sides of the experimental field. Amounts and times of fertilizer applications are shown in Table 1. Weed growth in plots was controlled using herbicides (atrazine and acetochlor) and removal by hand. Precipitation during maize growing season was 55.5, 112.1, 156.6, 212.9, and 70.7 mm from May to September in 2008 and 11.0, 39.2, 74.7, 26.5, and 65.0 mm in 2009. The total amount of rainfall during the maize growing season was 608 mm and 216 mm in 2008 and 2009, respectively. In addition, 43 mm of irrigation were applied on 2 July 2009 to avoid water shortage when plants were at elongation stage in the experiment.

**Table 1:** Fertilizer applications in 2008 and 2009.

**Year**	**Base fertilizer**			**V8**	**V12**			**Silking**			**Total (kg ha** ^−1^ **)**		
	**N**	**P**_**2**_ **O**_**5**_	**K**_**2**_ **O**	**N**	**N**	**P**_**2**_ **O**_**5**_	**K**_**2**_ **O**	**N**	**P**_**2**_ **O**_**5**_	**K**_**2**_ **O**	**N**	**P**_**2**_ **O**_**5**_	**K**_**2**_ **O**
2008	68	90	80	50	0	45	0	60	0	40	178	135	120
2009	60	135	80	100	40	0	40	0	0	0	200	135	120

N, P and K fertilizers were applied as urea, superphosphate and potassium chloride, respectively. Applications were made prior to sowing (base fertilizer), when the eighth leaf had emerged (V8), when the twelfth leaf had emerged and the ligule was visible (V12) and at silking. At V8, V12 and at the silking stage, fertilizers were applied as top dressing.

### Plant and soil sampling

The maize varieties used in this study differed in the duration of their phenological stages. Plants were harvested at silking (64 days after sowing, DAS, for BMY and JHH, 68 DAS for ZD2 and TK5, and 71 DAS for ND108 and ZD958) and at physiological maturity (111 DAS for BMY, JHH and ZD2, 116 DAS for TK5, and 130 DAS for ND108 and ZD958) in 2009, when over 50% of the plants showed a visible black layer at the kernel base. In 2008, plants were only harvested at silking. At harvest, five consecutive shoots were cut at the stem base, and two whole root systems were excavated within a soil volume of 50 cm (length) × 28 cm (width) × 40 cm (depth). Soil was removed from roots by gently washing and the use of a banister brush. The harvested shoot and root samples were chopped to a fine consistency, dried to a constant weight at 60°C, and ground into fine powders. Samples of ground, dried plant tissue were used to determine the total N content by a modified Kjeldahl digestion method [24]. The remaining digests were used for analyses of total P content (molybdovanadate method) by automated colorimetry [25] and total K content by flame photometry [26].

To study the spatial distribution of roots in the soil profile, a monolith method was employed [27]. Monoliths were excavated at silking in 2008 and at silking and maturity in 2009. One plant root system in each plot was excavated and, consequently, there were three replicates for each variety at each time point. This might be a minimal number of replicates considering the likely variation in root system architecture [28], but monolith excavation is both time consuming and labor intensive. This method was chosen because it reveals the actual distribution of roots in the soil volume and this can be compared directly with the local properties of the soil in the vicinity of these roots. Soil cubes with 10 cm edges (1000 cm^3^) were dug one by one in a soil volume of 60 cm (length) × 30 (width) cm to a depth of 60 cm. The total number of soil cubes sampled under each plant was 108. The soil of each cube of a monolith was crushed, and all of the visible roots in each soil cube were removed by hand in the field and placed in individual plastic bags labeled with the spatial coordinates. These roots were washed free of soil after transfer to the laboratory and then frozen at -20°C until the measurement of root length as described by Peng et al. [29]. The relative growth angles of shoot-borne roots of different maize varieties were estimated from the proportion of the root length in each soil cube excavated along the middle of the row for each soil horizon under plants at silking and at maturity in 2009.

After removing roots, the soil of each monolith cube was passed through a 3 mm sieve in the field. A portion of the sieved soil of each cube was then placed in a plastic bag labeled with the spatial coordinates and stored in the ice box. The bagged soil samples were extracted immediately after transfer to the laboratory with 0.01 mol L^−1^ CaCl_2_ solutions and analyzed for soil N_min_ (sum of NH_4_^+^-N and NO_3_^—^N) by continuous flow analysis (TRAACS 2000, Bran and Luebbe, Norderstedt, Germany) [30]. The remaining soil samples were then air-dried and extracted with 0.5 mol L^−1^ NaHCO_3_ for 0.5 h and then P concentration was analyzed colorimetrically according to the method described by Murphy and Riley [31]. The K concentration was analyzed following extraction with 1 mol L^−1^ NH_4_OAc by flame photometry according to the method described by Walker and Barber [26].

### Statistical analysis

**Fig 1:**
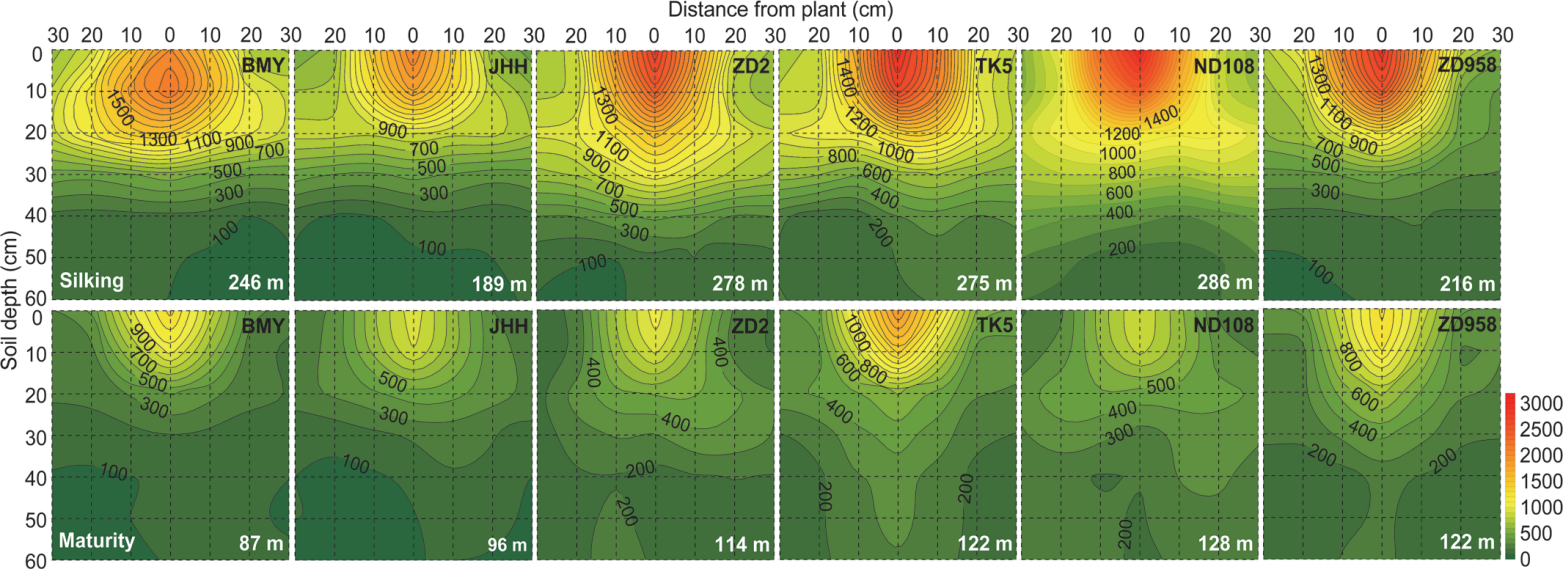
Two dimensional distribution of root length density (RLD, cm/3000 cm^3^) of different maize varieties at silking (the upper six panels) and maturity (the bottom six panels) in 2009. The roots were sampled in a soil volume of 60 cm (length, distance between rows of plants) × 30 cm (width, distance between plants within a row) × 60 cm (depth). Each 10×10 cm^2^ area was the projection of average RLD in the three 10×10×10 cm^3^ soil cubes across the width of the monolith. Maize varieties are indicated at the top right side and total root length per plant (m) in the whole 60×30×60 cm^3^ soil volume at both harvests are indicated at bottom right side in each panel, respectively. Data are mean values from 3 replicates of each genotype.

**Fig 2:**
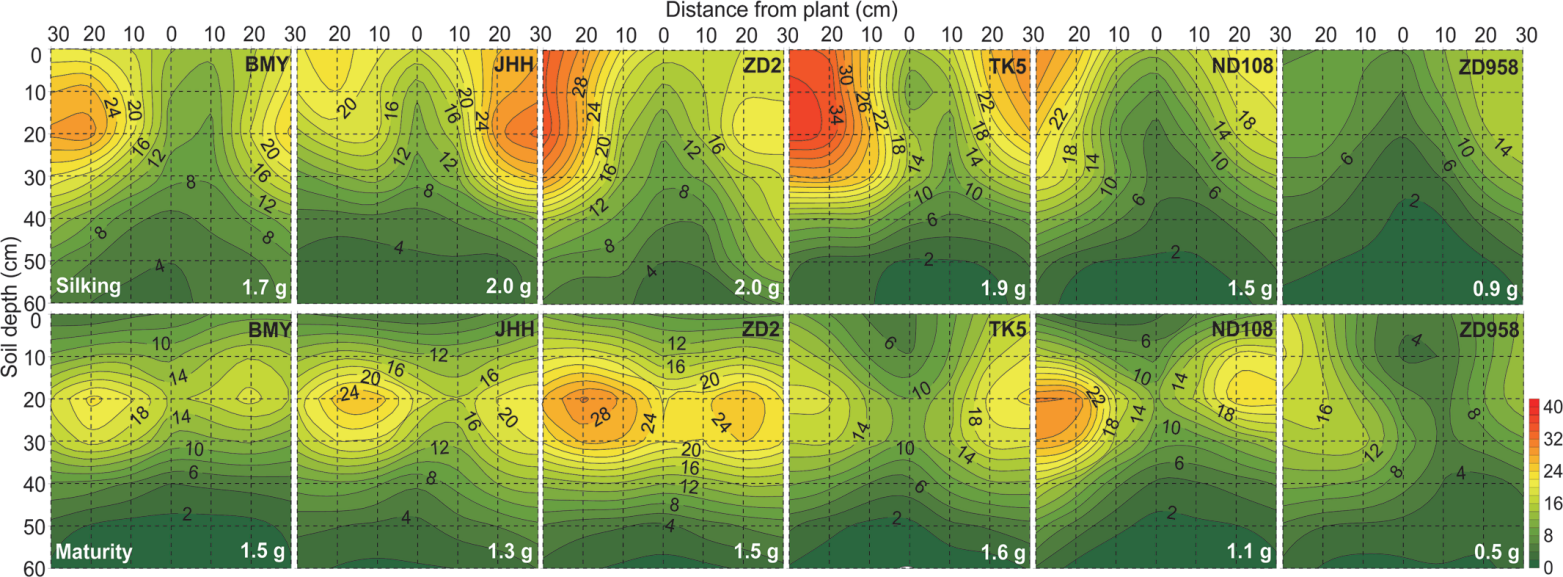
Two dimensional distribution of soil mineral nitrogen concentration (N_min_, mg/kg) of different maize varieties at silking (the upper six panels) and maturity (the bottom six panels) in 2009. The N_min_ was sampled in a soil volume of 60 cm (length)× 30 cm (width) × 60 cm (depth).Each 10×10 cm^2^ area was the projection of average N_min_ in the three 10×10×10 cm^3^ soil cubes across the width of the monolith. Maize varieties are indicated at the top right side and total soil residual mineral nitrogen content (g) in the whole 60×30×60 cm^3^ soil volume are indicated at the bottom right side in each panel, respectively. Data are mean values from 3 replicates of each genotype.

**Fig 3:**
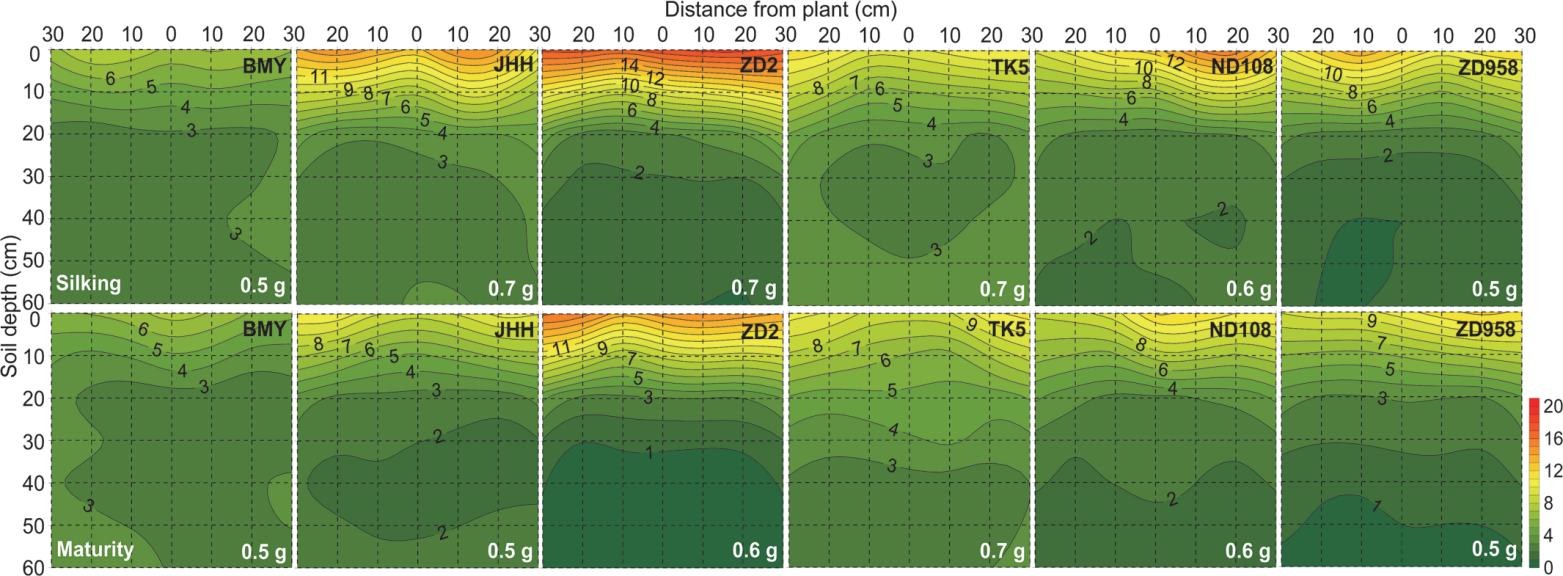
Two dimensional distribution of soil Olsen-P concentration (mg/kg) of different maize varieties at silking (the upper six panels) and maturity (the bottom six panels) in 2009. The Olsen-P was sampled in a soil volume of 60 cm (length)×30 cm (width)× 60 cm (depth). Each 10×10 cm^2^ area was the projection of average Olsen-P in the three 10×10×10 cm^3^ soil cubes across the width of the monolith. Maize varieties are indicated at the top right side and total soil residual Olsen-P content (g) in the whole 60×30×60 cm^3^ soil volume at both harvests are indicated at the bottom right side in each panel, respectively. Data are mean values from 3 replicates of each genotype.

**Fig 4:**
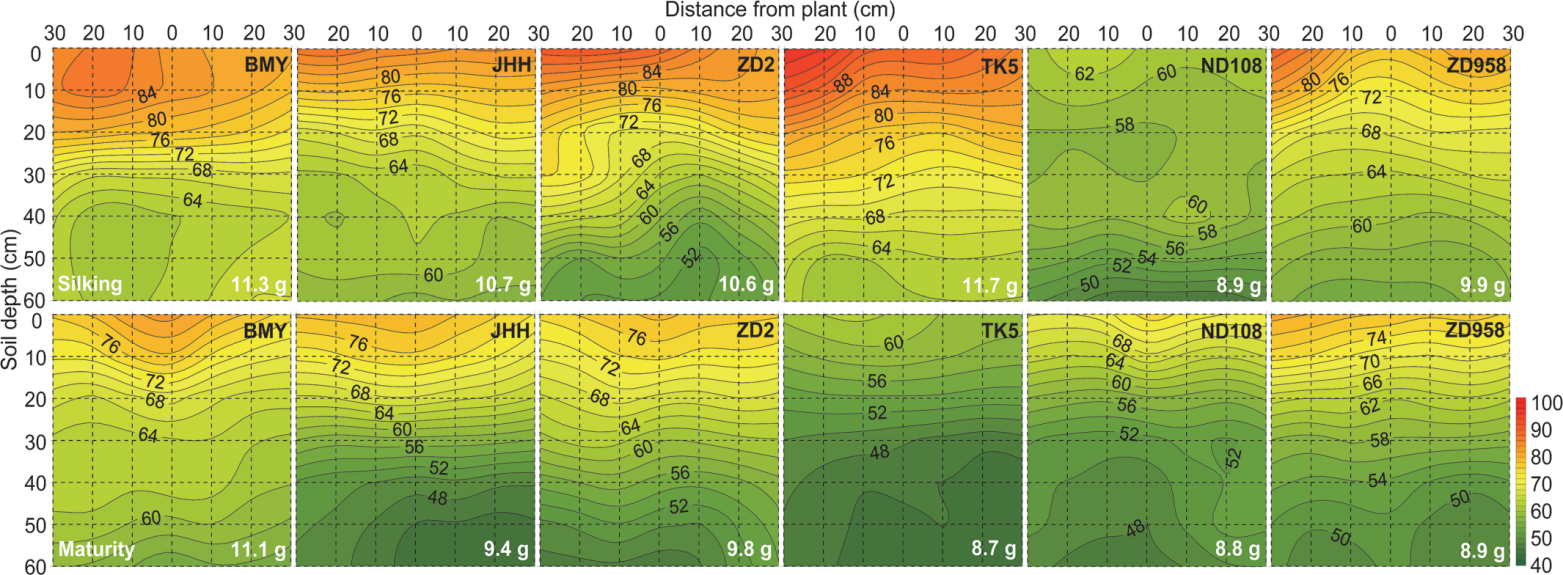
Two dimensional distribution of soil NH_4_OAc extractable K concentration (mg/kg) of different maize varieties at silking (the upper six panels) and maturity (the bottom six panels) in 2009. The NH_4_OAc extractable K was sampled in a soil volume of 60 cm (length)× 30 cm (width) × 60 cm (depth). Each 10×10 cm^2^ area was the projection of average NH_4_OAc extractable K in the three 10×10×10 cm^3^ soil cubes across the width of the monolith. Maize varieties are indicated at the top right side and total soil residual NH_4_OAc extractable K content (g) in the whole 60×30×60 cm^3^ soil volume are indicated at the bottom right side in each panel, respectively. Data are mean values from 3 replicates of each genotype.

**Fig 5:**
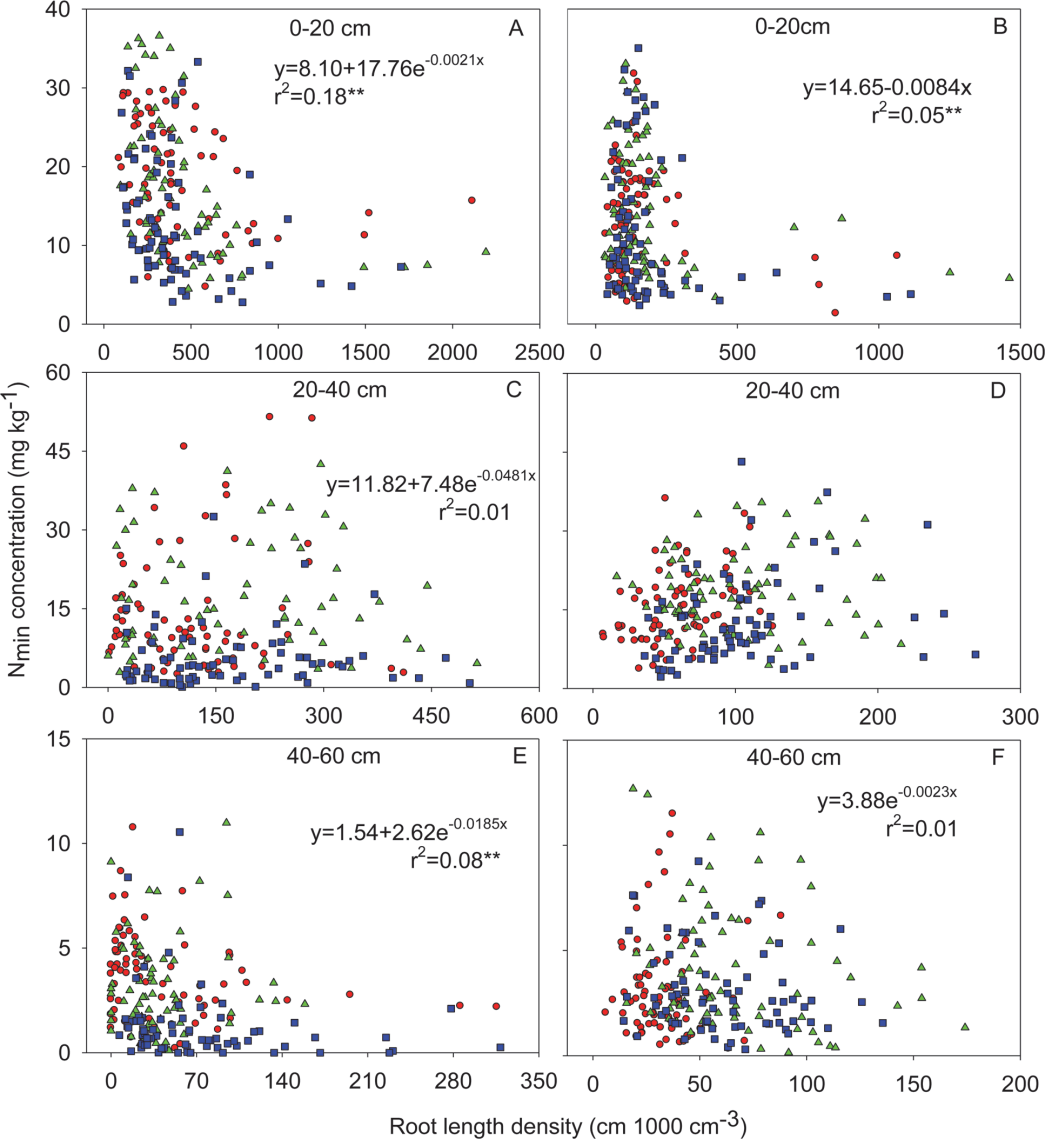
Relationships between soil N_min_ concentration and root length density in the 0–20 cm (A and B), 20–40 cm (C and D) and 40–60 cm (E and F) soil horizons at silking (A, C and E) and at maturity (B, D and F) in 2009. Each panel includes data from 216 cubes of soil, each of 1000 cm^3^, under six maize varieties of BMY and JHH in 1950s (red circles), ZD2 and TK5 in 1970s (green triangles) and ND108 and ZD958 in 1990s (blue squares). ** Significance at 0.01 probability level.

**Fig 6:**
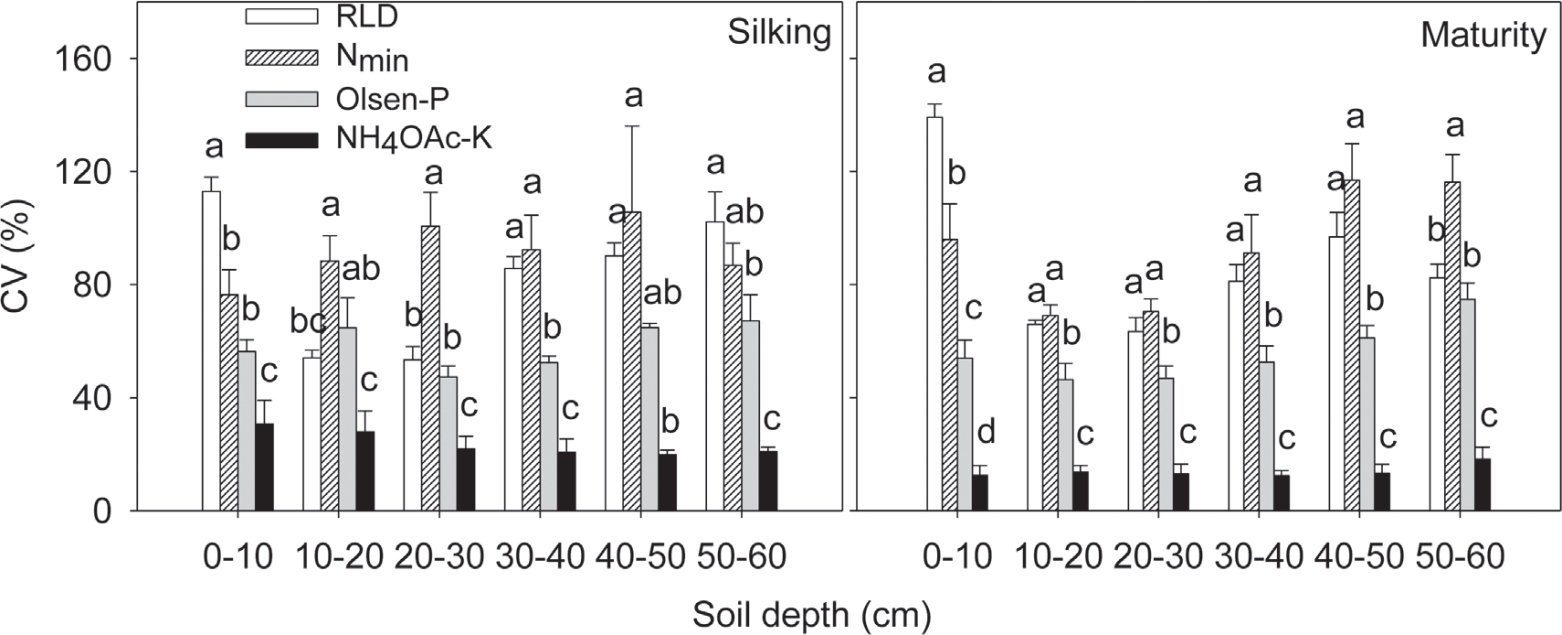
Coefficients of variation (CV, %) of root length density (RDL), soil N_min_, Olsen-P and NH_4_OAc-K concentration in each 10 cm soil horizon in excavated 60 cm (length) × 30 cm (width) × 60 cm (depth) soil monoliths at silking and maturity in 2009. Each column represents data of each 10 cm horizon from 108 soil monoliths (10×10×10 cm^3^), which was the product of 18 soil monoliths for each variety in a soil horizon times six maize varieties. Data are mean values from 3 replicates of each soil horizon. Different letters above the columns of each soil horizon indicate significant differences in coefficients of variation for RDL, N_min_, Olsen-P and NH_4_OAc-K (*P*<0.05).

Data from each harvest were analyzed separately by analysis of variance (ANOVA) using the SAS package 9.1 (SAS Institute, Cary, NC). Kriging interpolation was performed using Surfer 7.0 software (Golden Software, Inc. 2000) in the contour maps (Figs. 1, 2, 3, and 4; S1 Fig.). The relationships between N_min_ and RLD (Fig. 5) were best fitted using SigmaPlot statistical software. Differences in grain DW, shoot DW and shoot N, P and K concentrations between maize varieties (Table 2), in root length and root DW between maize varieties (Table 3), in soil N_min_, Olsen-P and NH_4_OAc-K in the soil volume beneath plants of different varieties and differences in the coefficient of variation (CV) in root length density, N_min_, Olsen-P and NH_4_OAc-K at a particular soil horizon (Fig. 6), in specific root length (S2 Fig.), in the percentage root length at particular locations in the soil below plants of different varieties (S4 Fig.) at a particular harvest were tested using PROC ANOVA. The least significant difference (LSD) was used to determine differences in means at a *P*<0.05 level of probability. The relationships between root length and grain yield or plant nutrient content at maturity (S3 Fig.) were determined using SigmaPlot statistical software (SigmaPlot 10.0, USA).

**Table 2:** Shoot and grain dry weight (DW; g m^−2^) and total N, P and K contents (g m^−2^) of different maize varieties at silking in 2008 and at silking and maturity in 2009.

**Years**	**Stage**	**Parameter**	**BMY**	**JHH**	**ZD2**	**TK5**	**ND108**	**ZD958**
2008	Silking	Shoot DW	693b	-	865b	801b	-	1168a
		Total N	12.4 b	-	13.8b	13.1 b	-	22.2 a
		Total P	1.2 c	-	1.6 b	1.2 b	-	1.5 a
		Total K	5.6 c	-	8.9 b	7.8 b	-	10.8 a
2009	Silking	Shoot DW	707bc	652 c	760bc	800 b	932 a	946 a
		Total N	13.3bc	11.0c	13.1bc	14.6 ab	16.6 a	15.9 a
		Total P	1.5bc	1.4 c	1.5bc	1.8 ab	2.0 a	1.8 a
		Total K	8.4b	7.4b	9.0ab	10.8 a	10.7 a	10.7 a
	Maturity	Shoot DW	1295bc	1074c	1371 b	1763 a	2037 a	2006 a
		Grain DW	572bc	468 c	613 b	932 a	1013 a	1069 a
		Total N	16.3 b	13.4 b	15.8 b	20.5 a	22.7 a	20.9 a
		Total P	2.0 b	1.7 b	2.1 b	3.4 a	3.4 a	2.7 a
		Total K	6.9 c	6.4c	8.8 b	10.8 ab	11.2 a	12.5 a

BMY and JHH are early senescing varieties released in the 1950s; ZD2 and TK5 are two intermediate senescing hybrids released in the 1970s; ND108 and ZD958 are two currently popular stay-green hybrids. Values in rows followed by different letters represent significant differences between varieties (*P*<0.05) at a particular harvest. Values are means of three replications.

**Table 3:** Total root length, root DW and soil available nutrient content (Soil N_min_, Olsen-P and NH_4_OAc-K) in a soil volume of 60×30×60 cm^3^ (length × width × depth) under different maize varieties at silking in 2008 and at silking and maturity in 2009.

**Years**	**Stage**	**Parameters**	**BMY**	**JHH**	**ZD2**	**TK5**	**ND108**	**ZD958**
2008	Silking	Root length (mplant^−1^)	263 a	-	242 a	312 a	-	302 a
		Root DW (gplant^−1^)	9.5 b	-	11.7 ab	14.4 ab	-	17.2 a
2009	Silking	Root length (mplant^−1^)	246 ab	189 b	278 a	275 ab	286 a	216 ab
		Root DW (gplant^−1^)	7.2 b	6.5 b	7.4 b	9.8 ab	11.0 a	12.6 a
		Soil N_min_ (g)	1. 7 a	2.0 a	2.0 a	1.9 a	1.5 ab	0.9 b
		Soil Olsen-P (g)	0.5 a	0.7 a	0.7 a	0.7 a	0.6 a	0.5 a
		Soil NH_4_OAc-K(g)	11.3 a	10.7 a	10.6 a	11.7 a	8.9 a	9.9 a
	Maturity	Root length (mplant^−1^)	87c	96 c	114 b	123 ab	128a	122 ab
		Root DW (gplant^−1^)	4.1 b	4.0 b	5.4 b	9.2 a	8.8 a	9.0 a
		Soil N_min_ (g)	1.5 a	1.3 ab	1.5 a	1.6 a	1.1 ab	0.7 b
		Soil Olsen-P (g)	0.5 a	0.5 a	0.6 a	0.7 a	0.6 a	0.5 a
		Soil NH_4_OAc-K(g)	11.1a	9.4 ab	9.8 ab	8.7 b	8.8 b	8.9 b

Values followed by different letters in rows represent significant differences among varieties (*P*< 0.05) at a particular harvest. Values are means of three replications.

## Results

### Plant growth and nutrients uptake

New maize varieties (ND108 and ZD958) had accumulated more dry weight (DW) and nutrients (N, P and K) in their shoots than older varieties at silking (Table 2). Differences in shoot DW and plant nutrient content between the new and older varieties increased between silking and maturity. In 2009, the percentage increase in shoot DW between silking and maturity was 83%, 65%, 119% and 112% for two older varieties BMY, JHH, and two new ones ND108 and ZD958, respectively. Similar changes can be observed for plant nutrient content. Because of their larger shoot DW, the new varieties had greater grain yield than the older varieties (Table 2).

### Root spatial distribution in the 0–60 cm soil profile

The distribution of roots of different maize varieties in a soil volume of 60 cm (length) × 30 cm (width) × 60 cm (depth) was investigated at silking in 2008 and at silking and maturity in 2009. These distributions are presented as projected two dimensional RLD across the width of a monolith (Fig. 1; S1 Fig.). The majority of the total root length of all maize varieties was present in the topsoil (0–30 cm) at both silking and maturity. In 2009, for example, over 80% of the total root length of all maize varieties was present in the 0–30 cm soil horizon at silking and 65–89% at maturity. The RLD in the next horizons (30–60 cm) decreased with increasing soil depth for all maize varieties (Fig. 1; S1 Fig.). RLD in deeper 30–60 cm soil horizons was similar among all varieties at silking. However, new varieties had greater RLD in the same soil horizon than the older varieties and thus higher total root length at maturity (Fig. 1), because of a less decrease in total root length in older varieties after silking. Horizontally, RLD was greatest in the soil directly under the base of the stem and decreased with increasing distance from the stem base in the top 0–20 cm horizon.

Root DWs of new varieties (ND108 and ZD958) were larger than those of older varieties at silking and the relative difference in root DW between the new and older varieties increased after silking (Table 3). In 2009, the percentage decrease in root DW between silking and maturity was 43%, 39%, 20% and 27% for BMY, JHH, ND108 and ZD958, respectively (Table 3). In contrast to root DW, the total root length of new varieties did not differ significantly from that of older varieties at silking, this was consistent with the results that new varieties had lower specific root length (the ratio of root length to root DW, m g^−1^) than older ones at silking and maturity, showing a higher proportion of thicker roots in new varieties (S2 Fig.). However, new varieties had significantly greater total root length than the oldest varieties at maturity (Table 3), especially in the 0–30 cm soil horizon (Fig. 1). Positive relationships were observed between total root length and plant N, P and K contents at maturity and grain yield (S3 Fig.).

The growth angles of shoot-borne roots were not determined directly in this study. However, to assess whether new varieties had steeper roots than older varieties, the proportion of the root length in each soil cube excavated along the middle of the row was determined for each soil horizon for plants at silking and at maturity (S4 Fig.). Similar proportions of the total root length in soil cubes located at the same distance from the stem axis in all soil horizons suggested that there were no differences among the maize varieties studied here in growth angles of shoot-borne roots.

### Spatial distribution of soil available N, P and K in the 0–60 cm soil profile

The soil mineral nitrogen (N_min_) concentration (Fig. 2; S1 Fig.), Olsen-P (Fig. 3; S1 Fig.) and NH_4_OAc extractable K (Fig. 4; S1 Fig.) were greatest in the top soil and decreased with increasing soil depth, regardless of whether they were assessed at silking or maturity.

There was a negative relationship between soil N_min_ concentration and RLD both at silking and maturity, and soil N_min_ was significantly lower where RLD was highest, especially under the plant stem axis in the 0–20 cm horizon in both years (Figs. 1, 2, 5; S1 Fig.). In 2009, the total N_min_ in the soil when new varieties (ND108 and ZD958) were grown was less than that in the soil when older varieties were grown at both silking and maturity (Fig. 2). This was consistent with the greater N accumulation of new varieties (Table 2). Furthermore, soil N_min_ concentration in the 0–10 cm horizon was dramatically reduced at maturity compared with that at silking in 2009 (Fig. 2).

In contrast to the data for N_min_, there was no obvious relationship between RLD and either Olsen-P or NH_4_OAc extractable K in the soil profile for any maize variety at any harvest (Figs. 2, 3, and 4; S1 Fig.). The horizontal variation of Olsen-P and NH_4_OAc extractable K in each soil layer was also less than that of RLD and soil N_min_ (Fig. 6). The horizontal variation (expressed as coefficients of variation, CV, %) was greatest for RLD (53.4%–113.0%) and soil N_min_ (76.4%–105.6%) and least for NH_4_OAc extractable K (19.9%–30.7%) (Fig. 6). Despite greater P and K accumulation in new varieties than older varieties (Table 2), there were no differences between varieties in the total Olsen-P and NH_4_OAc extractable K remaining in the soil after their cultivation (Table 3).

## Discussion

### New maize varieties had the similar root total length and vertical distribution but accumulated more nutrients at silking compared with the old ones

Since the maize varieties used in this study differed in the duration of their phenological stages, they were harvested different times after sowing at silking and maturity. However, the larger shoot DW and grain yield of new varieties compared with old ones are because of not only a longer growth season in the new varieties, but also larger total leaf area and longer green leaf duration (stay-green) [1, 23, 32, 33]. Although new varieties take up more N, P and K after silking than old varieties, large amounts of N, P and K accumulated pre-silking are remobilized and exported from vegetative tissues in all varieties during grain filling. The export of N, P and K from leaves is similar in all these varieties [23].

Despite having a larger root DW (Table 3; [2]), new maize varieties had similar total root lengths and vertical root distributions to older varieties at silking (Table 3; Fig. 1; S1 Fig.), when the root system of maize is largest [14], indicating that new varieties had higher proportion of thicker roots than older ones (S2 Fig.; [34]). Thicker roots have several advantages: increased root lengths or growth rates, increased water transport [35, 36] and root penetration index [37], and improved lodging resistance [38]. Agricultural top soils are rich in the macro- and micro-elements necessary for plant growth, which comes from long-term fertilizer applications, mineralization of organic matter, and degradation of plant residues in the 0–30 cm soil horizon [39]. It is therefore reasonable for the root system of maize to proliferate in the top soil to access the soil mineral nutrients to meet plant demand.

Although both new and older maize varieties had similar total root lengths at silking, new varieties accumulated more mineral nutrients than older varieties. The amounts of mineral nutrients taken up by plants with an ample supply are often determined by plant demand, rather than simply by the size of their root system [29, 40–45]. In addition, there is often redundant growth of plant roots, which has been interpreted as a precautionary investment of surplus assimilates in the ‘over-growth’ of roots [46]. For example, Robinson et al. [47] estimated that only 11% and 3.5% of the total root length of wheat contributed to nitrate uptake in a pot experiment in which plants were supplied with limited or sufficient N, respectively. However, plant root plasticity in response to the heterogeneous distribution of soil resources is not “redundant” growth. Root plasticity is manifest in the ability of plants to optimize root system architecture for resource acquisition under diverse environmental conditions in response to the heterogeneous distribution of soil resources [48–50]. For example, plants proliferate a lot of lateral roots into the nitrate-rich patches, which helps plant to take up more nitrate or to take it up faster [51–53].

### New maize varieties had greater post-silking total root length than older ones

At maturity, new maize varieties had greater total root length than older varieties (Table 3). The results indicated that old varieties lost more root length than the new ones after silking. This was a consequence of reduced root mortality of old roots or vigorous growth of new roots, especially of roots in the top (0–30 cm) soil horizon, after silking (Fig. 1; S1 Fig.). New varieties had a greater post-silking demand for mineral nutrients than older varieties, and the delayed root mortality or vigorous growth of new roots of new varieties could improve post-silking uptake of nutrients (Table 2). Stay-green leaves of new varieties allow more carbohydrate allocation to roots and have a positive effect on root growth, which in turn promotes nutrient uptake [54], thereby, supports higher grain yield. Indeed, the acquisition of mineral nutrients and grain yields were positively related with the total root length at maturity (S3 Fig.).

Lateral roots constitute the majority of the total root length [29] and are responsible for the acquisition of mineral nutrients [12, 55]. The relationship between root length and distance from the plant shoot axis along the middle row was used to assess the root growth angle at silking and maturity. The same horizontal root length distribution among varieties in all soil horizons suggested similar growth angles of shoot-borne roots (S4 Fig.). However, it is possible that the relatively large size of soil cubes used in the present study might not distinguish small differences in root length distribution and, thus, small differences in root growth angles among varieties. The shoot-borne roots angles could be directly measured by protractor or phenotyped by shovelomics with visual score method [56]. Compared to them, we, to some extent, missed precision by comparing root length distribution.

The experiments reported here were conducted with the same plant density. It is known that the size of the maize root system is reduced with increasing plant density [15, 57]. Further experiments are required to determine whether root growth angle of maize plants changes when they were grown at higher plant densities. It has been reported that maize leaf orientation is sensitive to plant density, and this has been interpreted as a shade avoidance reaction controlled by the red:far-red ratio of light within the canopy [58]. Plant root systems might have similar architectural responses to increased root competition as planting density is increased.

### **Variation in N**_min_ **was larger than variation in available P and K in soil monoliths**

There was a significantly negative relationship between RLD and soil N_min_ concentration in the top 0–20 cm horizons both at silking and maturity (Figs. 1, 2, 5; S1 Fig.). This observation suggests that the size and spatial distribution of the root system in the soil profile is important, not only for the uptake of nutrients with low mobility in the soil, such as P and K, but also for nitrate uptake, despite the greater mobility of nitrate in the soil compared to those of phosphate and K. Interestingly, the net accumulation of N by maize plants of all varieties from silking to maturity exceeded the reduction of soil N_min_ in whole soil profile during the same period (Tables 2, 3). This suggests that N mineralization and/or N uptake from deeper soil layers might contribute to the plant’s N requirement. In higher plants, 20%–60% of photosynthetic carbon is allocated below-ground, of which the estimates of rhizodeposition can comprise up to 40% in annual plants. Carbon rhizodeposition by growing roots enhances the microbial turnover of soil organic carbon in the rhizosphere [59]. Differing from the bulk soil, where microbial growth is C-limited, microbial growth in the rhizosphere is N-limited, which increases microbial demand for N [59, 60]. Considering the dynamics of root and microbial turnover, the plant root have repeated opportunity to compete for N for its longer life-spans (order of weeks to months), while the turnover of microbial biomass in the rhizosphere is rapid (order of hours to days) [60].

On the single root basis, reduction of the nitrate concentration at the root surface is less than that of P and K [16, 18]. On the root zone basis under the present field condition, however, neither the Olsen-P nor the NH_4_OAc extractable K was reduced as dramatically as N_min_ in volumes of the soil with high RLD (Figs. 1, 2, 3, and 4; S1 Fig.). As a consequence, the spatial variation of soil N_min_ in each soil horizon was larger than that of Olsen-P and NH_4_OAc extractable K in the soil (Fig. 6). This might be related to the relatively large size of the soil cubes removed from the soil monoliths, if the depletion of Olsen-P and NH_4_OAc extractable K in the soil was restricted to a smaller volume of soil surrounding the root than the depletion of N_min_. Alternatively, uptake of N relative to its supply might have been greater than that of P and K. All maize varieties accumulated more N than P or K.

## Conclusion

Despite having larger total root DW, new maize varieties (ND108 and ZD958) had similar total root length, root vertical distribution in the 0–60 cm soil profile and root growth angles to older maize varieties at silking when grown under the experimental conditions described here. Thus, the greater nutrient accumulation and grain yield of new maize varieties was not predicated based on greater total root length than older varieties at silking. It is, perhaps, related to the regulation of nutrient uptake capacity of the root by plant demand. Nevertheless, since most of the soil resources are distributed in the topsoil, the proliferation of roots in the topsoil before silking and the maintenance of roots in the topsoil after silking ensure that new maize varieties take up sufficient soil resources to support their greater nutrient demand and higher grain yield than older varieties. Larger root DW in new maize varieties is also beneficial for lodging resistance at high plant density [1, 61]. Although nitrate is relative mobile in the soil, both horizontally and vertically, a negative relationship between RLD and soil N_min_ concentration was observed in the top 0–20 cm horizon for all varieties and the variation in soil N_min_ concentration was greater than that of Olsen-P and NH_4_OAc extractable K throughout the soil profile, suggesting that root spatial distribution in the soil is important for N uptake by maize. Because of relative larger variation in the field conditions compared with that under the controlled conditions, it is better to have more replicates for experiments carried in the field. Future studies are required to determine the physicochemical changes in the rhizosphere as a consequence of root activity. Root growth and/or turnover after silking must be an important factor influencing the rhizosphere processes.

## Supporting Information

S1 Fig Two dimensional distribution of root length density (cm/3000 cm^3^), soil mineral nitrogen concentration (mg/kg), soil Olsen-P concentration (mg/kg) and soil NH_4_OAc extractable K concentration (mg/kg) of different maize varieties sampled at silking in 2008. Root length density, soil mineral nitrogen concentration, soil Olsen-P concentration and soil NH_4_OAc extractable K concentration were shown in row from the top to bottom respectively, in a soil volume of 60 cm (length,) × 60 cm (depth) × 30 cm (width). Each 10 ×10 cm^2^ area was the projection of three 10×10×10 cm^3^ soil cubes. Maize varieties were indicated at the top right side. Total root length per plant (m), soil mineral nitrogen content (g), soil Olsen-P content (g) and soil residual NH_4_OAc extractable K content (g) in the whole 60×30×60 cm^3^ soil volume were indicated at bottom right side in each panel, respectively. *n* = 3.
(TIF)

S2 Fig Specific root length of roots in each 10 cm soil layer of six maize varieties at silking in 2008, and at silking and maturity in 2009. Specific root length means the ratio of root length to root dry weight. Different letters above the bars within the same soil layer indicate significant differences among maize varieties (*P*<0.05). Data are mean values from 3 replicates of each genotype. Error bars denote the standard deviation.
(TIF)

S3 Fig Relationships between total root length and grain yield (A), plant N content (B), plant P content (C) and plant K content (D) in maize varieties (● BMY,○ JHH, ▲ ZD2, △ TK5, ■ ND108 and □ ZD958) at maturity in 2009. * and ** represent significance at 0.05 and 0.01 probability level, respectively.
(TIF)

S4 Fig Percentage of root length of six maize varieties in 1000 cm^3^ cubes in the middle row of each soil horizon n from an excavated 60 cm (length) × 30 cm (width) × 60 cm (depth) soil monolith at silking (A-F) and maturity (G-L) in 2009. Different letters above the bars indicate significant differences between maize varieties in a 1000 cm^3^ cube (*P*<0.05). Data are mean values from 3 replicates of each genotype. Error bars denote the standard deviation.
(TIF)
