# Supplementary figures and images for: Maize Varieties Released in Different Eras Have Similar Root Length Density Distributions in the Soil, Which Are Negatively Correlated with Local Concentrations of Soil Mineral Nitrogen
Source: PLoS One. 2015 Mar 23;10(3):e0121892. doi: 10.1371/journal.pone.0121892 (PMC4370465; doi:10.1371/journal.pone.0121892)

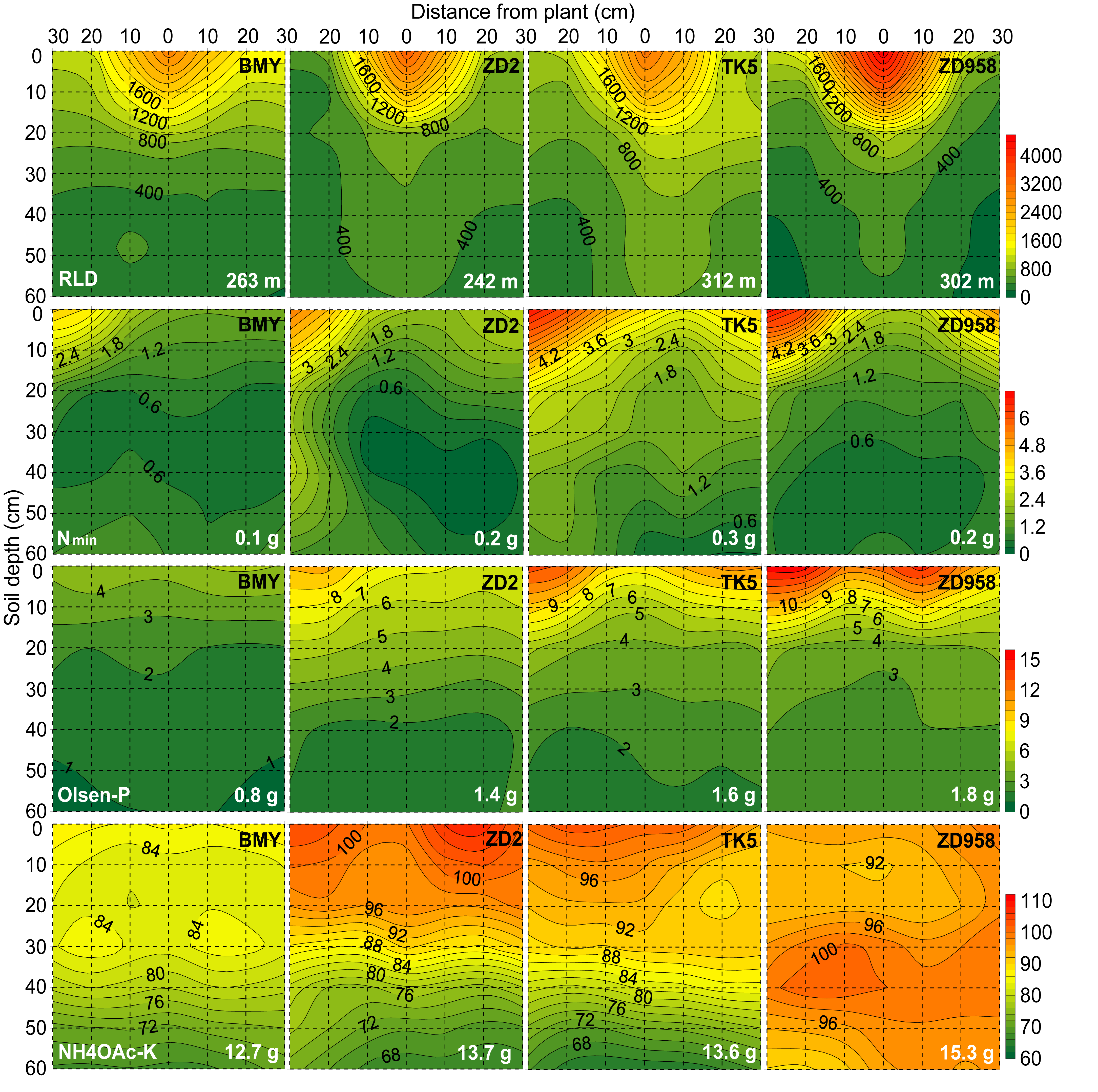

Supplement: S1 Fig — Root length density, soil mineral nitrogen concentration, soil Olsen-P concentration and soil NH4OAc extractable K concentration were shown in row from the top to bottom respectively, in a soil volume of 60 cm (length,) × 60 cm (depth) × 30 cm (width). Each 10 ×10 cm2 area was the projection of three 10×10×10 cm3 soil cubes. Maize varieties were indicated at the top right side. Total root length per plant (m), soil mineral nitrogen content (g), soil Olsen-P content (g) and soil residual NH4OAc extractable K content (g) in the whole 60×30×60 cm3 soil volume were indicated at bottom right side in each panel, respectively. n = 3. (TIF) [file pone.0121892.s001.tif]

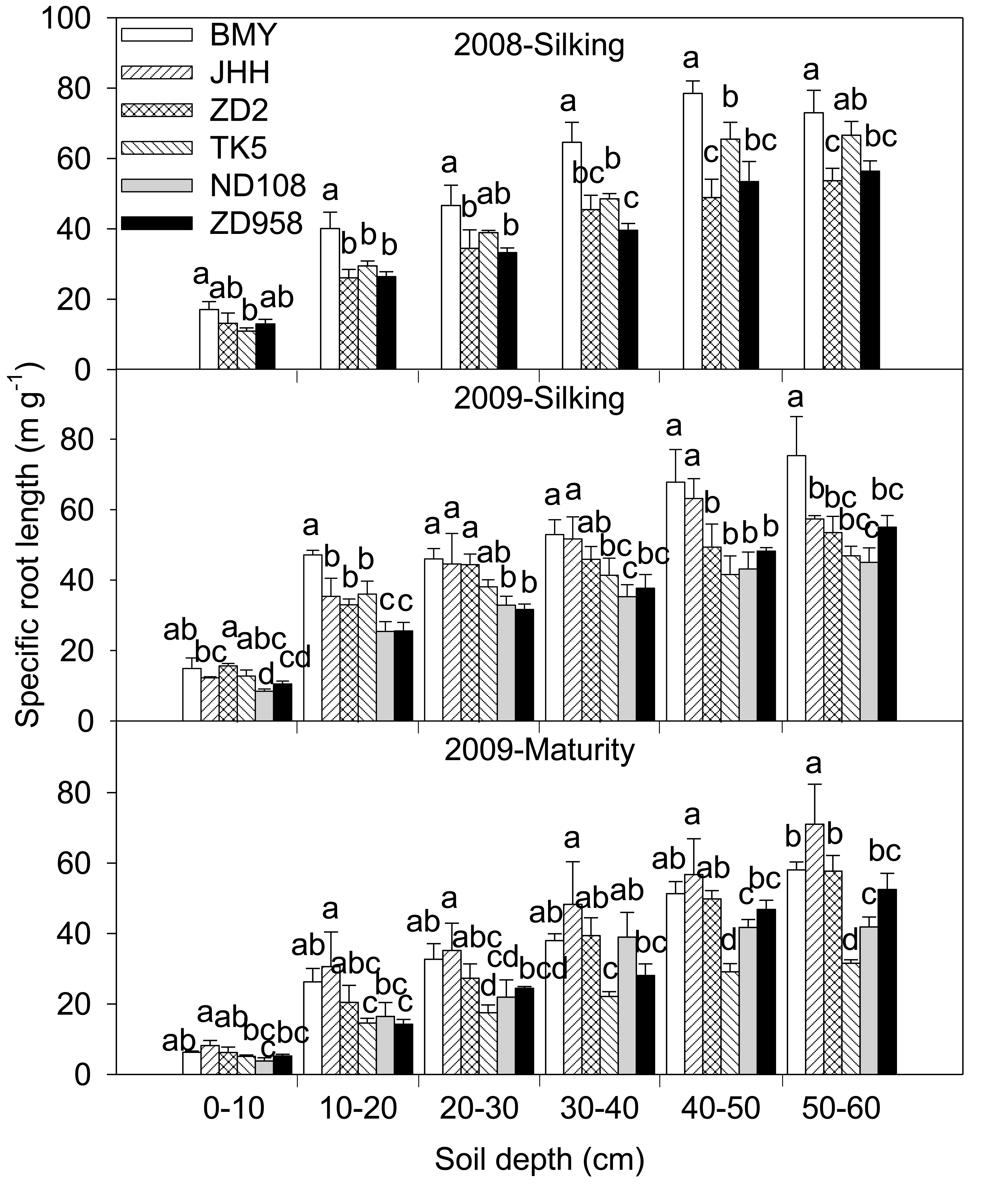

Supplement: S2 Fig — Specific root length means the ratio of root length to root dry weight. Different letters above the bars within the same soil layer indicate significant differences among maize varieties (P<0.05). Data are mean values from 3 replicates of each genotype. Error bars denote the standard deviation. (TIF) [file pone.0121892.s002.tif]

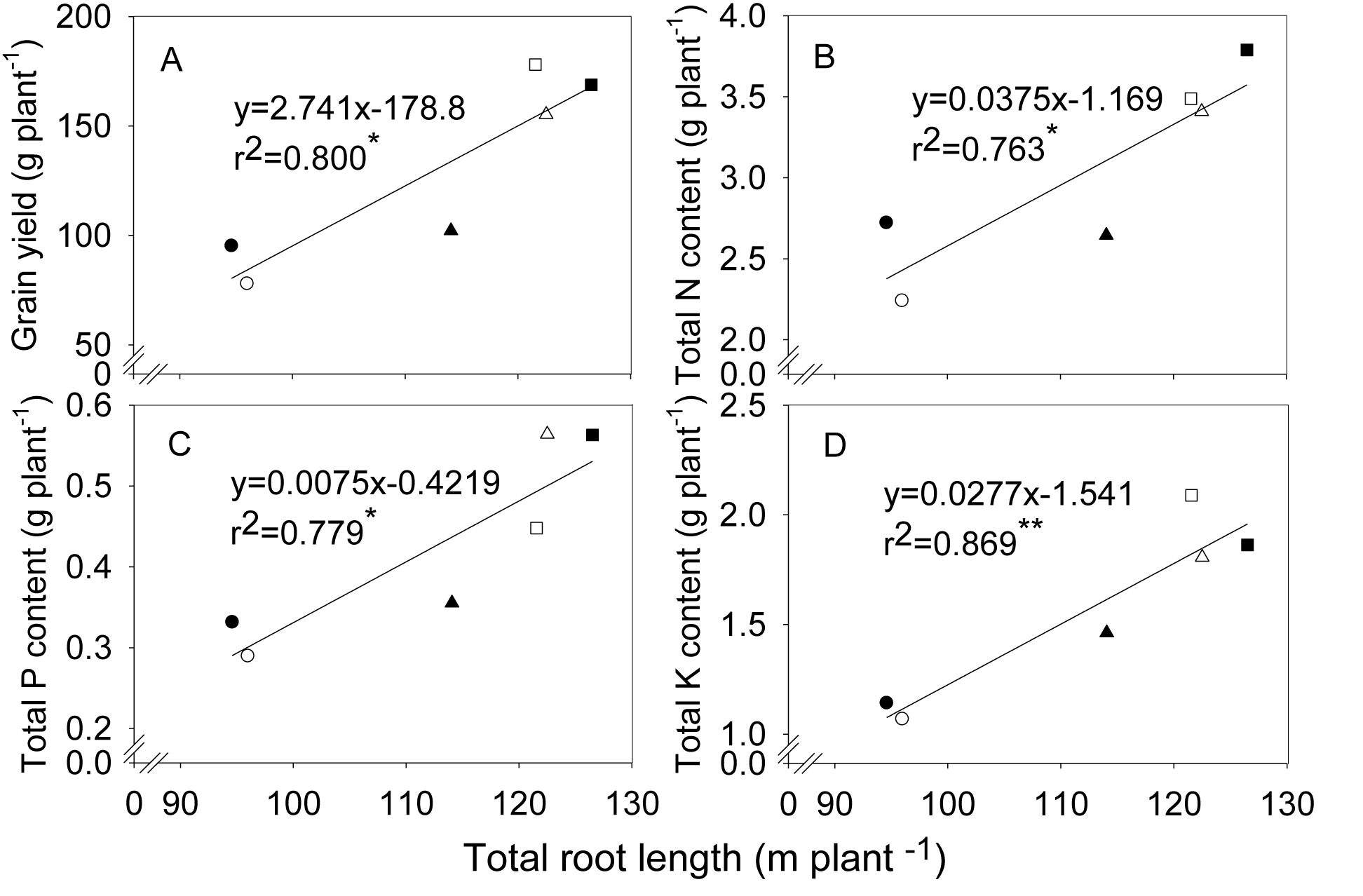

Supplement: S3 Fig — * and ** represent significance at 0.05 and 0.01 probability level, respectively. (TIF) [file pone.0121892.s003.tif]

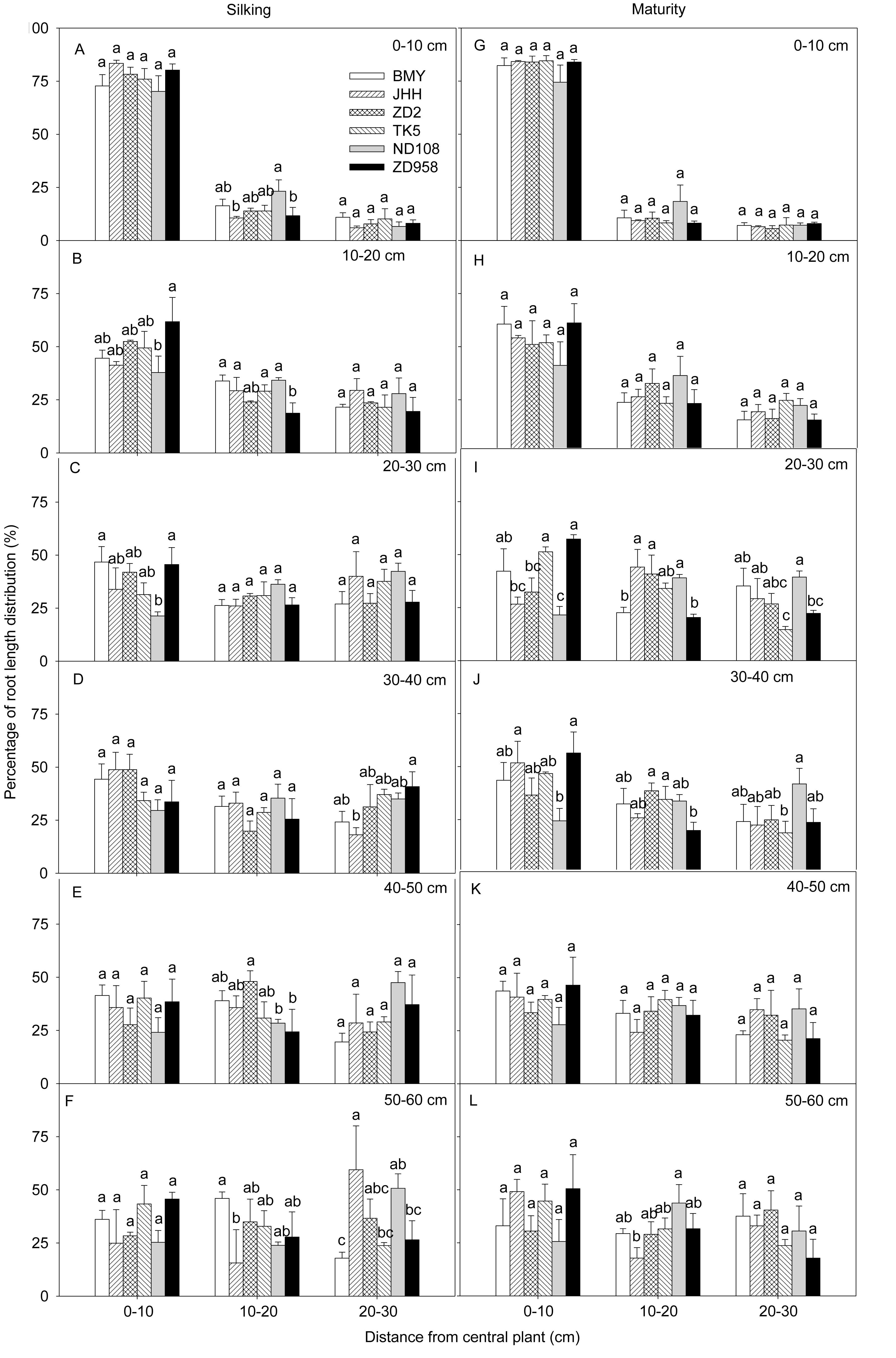

Supplement: S4 Fig — Different letters above the bars indicate significant differences between maize varieties in a 1000 cm3 cube (P<0.05). Data are mean values from 3 replicates of each genotype. Error bars denote the standard deviation. (TIF) [file pone.0121892.s004.tif]
